# Interviews: A collection of stories and perspectives about the past and future of GnRH research

**DOI:** 10.1111/jne.13140

**Published:** 2022-05-28

**Authors:** Michael N. Lehman

**Affiliations:** ^1^ Brain Health Research Institute and Department of Biological Sciences Kent State University Kent Ohio USA

## 1 INTRODUCTION

As an endpiece to this Special Issue celebrating the 50th anniversary of the discovery of GnRH, we asked a group of luminaries in this area of neuroendocrine research to consider the past and future of GnRH research. The following questions were not meant to cover all possibilities, but rather to provide an interesting range of perspectives, looking backward at 50 years of research and forward toward future challenges and opportunities in the field. I am grateful to the respondents, all of whom agreed to be identified informally, for their very interesting and illuminating answers to these questions and thank them for sharing their views and stories with us.

## 2 WHAT DO YOU THINK HAS BEEN THE MAIN BREAKTHROUGH OF THESE 50 YEARS OF GnRH RESEARCH?

### 2.1 Bill Crowley

There are several major basic and clinical discoveries that have represented enabling technologies that taken together have generated major conceptual or scientific breakthroughs in GnRH over the past five decades. These fall into five major categories:

1. The availability of GnRH derived cell lines using SV40 viral transformations such as the GT1‐7 (Mellon), Gn11 (Radovick), etc. These single progenitor cell lines have been critical in identifying our evolving understanding of the complexity of the intracellular genetic and biochemical pathways involved in GnRH transcription, biosynthesis, and secretion. They have all been found human disease counterparts that represent errors in them with clinical consequences.

2. New techniques for identifying and studying neuronal fate specification and cellular tagging that have enabled developmental tracking and definition of the neuronal “dendrons” that govern GnRH section and interneuronal synapses and communications across the CNS.

3. The identification of several human disease models of GnRH deficiency such as Isolated GnRH Deficiency and Kallmann Syndrome, that have allowed identification of ~4 dozen genes in which mutations can cause congenital defects in GnRH biosynthesis, secretion, and/or action.

4. A derivative of this novel gene discovery effort was the surfacing of the kisspeptin pathway by French investigators that was confirmed by our group. This single gene discovery unearthed yet another level to the hierarchical control of GnRH and offered a mechanism by which other environmentally sensitive genes governing reproduction during starvation was identified.

5. The medical contribution of important novel therapeutics for GnRH agonists. GnRH agonist use in humans was pioneered in children with precocious puberty in the *New England Journal of Medicine* (*NEJM*) in 1980 and still they remain the treatment of choice for this disorder now 40 after their publication in *NEJM*. These studies in turn laid the scientific framework for their use to induce a reversible biochemical castration and sex steroid withdrawal not only in precocity. They laid the scientific groundwork for their current use in prostate and breast cancer, endometriosis, IVF and uterine fibroids. They now represent a robust $3.5B annual expenditure for the mitigation/treatment of serious illnesses.

### 2.2 Bob Goodman

I would pick the KNDy model for GnRH pulse generation. This undoubtedly reflects some bias on my part, but I think a strong objective case can be made for this. Specifically, the existence of episodic GnRH release has been recognized since before GnRH was identified and attempts to determine the underlying mechanisms have been a major focus of the field since then. This also has as a corollary, the discovery of kisspeptin, which was another major breakthrough, and was a necessary precursor for the development of this model.

### 2.3 Fred Karsch

Being a physiologist, I think the most important breakthrough in 50 years of GnRH research has been the discovery that GnRH secretion is pulsatile and that optimal secretion of gonadotropic hormones from the anterior pituitary gland requires this episodic pattern of GnRH release. Once its structure was identified and synthetic GnRH became available, studies in the 1970s in rhesus monkeys revealed that intermittent delivery of GnRH for a few minutes each hour for long periods of time potently stimulates LH and FSH secretion, whereas prolonged continuous delivery of GnRH fails to support gonadotropin secretion and causes the pituitary to become refractory to GnRH. Complementary observations came several years later, once procedures were developed to monitor the time course of GnRH secretion directly by high frequency sampling of pituitary portal blood. Studies in sheep demonstrated that, apart from the preovulatory GnRH surge, the hormone is secreted as abrupt bursts, lasting but a few minutes, with little or no secretion between these bouts of release.

Why do I single this out as the most important breakthrough? The finding that intermittent, as opposed to continuous, stimulation of the pituitary is critical has fueled an entire research field to explore the pulsatile pattern of LH (and in some cases GnRH) secretion in various physiological and pathological conditions, and how both frequency and amplitude of pulses change throughout the estrous/menstrual cycle and in relation to alterations in fertility, for example during sexual maturation, hypothalamic amenorrhea, and seasonal shifts in reproductive function. Complementary research determined how GnRH pulse frequency and amplitude are regulated by factors in the internal and external environments (gonadal steroid feedback, malnutrition, social cues, daylength, stress, etc.) and that this regulation is critical to fertility. In essence, the discovery of the pulsatile mode of GnRH release and its functional significance have provided a conceptual framework for our current understanding of how the brain controls reproduction.

### 2.4 Jon Levine

I believe that the “main breakthrough” actually consists of a series of breakthroughs that together have provided an understanding of GnRH pulse generation and its roles in reproductive physiology and disease. Each of these built on prior findings in an exquisite progression of insights that led to our current understanding of the cellular basis of GnRH pulsatility. Early studies in gonadectomized monkeys, gonadectomized rats, and women with menstrual disorders revealed that peripheral LH or FSH levels fluctuate greatly in samples drawn >1h apart. Shortly thereafter, these seemingly random fluctuations were shown to be regular, “circhoral oscillations” (pulses) in ovariectomized monkeys and castrated rats, demonstrating the existence of a mechanism by which LH release occurs in a pulsatile manner. Ernst Knobil presciently hypothesized that “…these discharges may be due to intermittent signals from the central nervous system…which, in turn, result in an increased production of LH releasing factor and the discharge of LH”. By 1976, Michel Ferin and colleagues characterized pulsatile GnRH release in the hypophysial portal vasculature of anesthetized monkeys, and thereafter pulsatile GnRH release was measured in unanesthetized, ovariectomized ewes by portal blood collection and push‐pull perfusion of the median eminence and shown to be directly associated with pulsatile LH patterns in the periphery. It would take several more decades to determine whether GnRH pulsatility is an intrinsic function of GnRH neurons, or if it is primarily driven by afferent signals originating from non‐GnRH cell group(s). The latter mechanism was suggested by the demonstration that GnRH release can occur in a pulsatile manner from mediobasal hypothalamic fragments devoid of GnRH soma. Ultimately, the identification of kisspeptin neurons as the major afferent network controlling GnRH release, the demonstration that Kiss1 receptors in GnRH neurons mediate stimulation of GnRH release, and the characterization of synchronized activation of arcuate Kiss1 neurons in association with pulsatile LH secretion, confirmed the hypothesis that arcuate Kiss1 neurons function as a GnRH pulse‐generating network.

The importance of this series of breakthroughs is evidenced by the myriad discoveries that sprang from this work. These included demonstration of the dependence of gonadotropin secretions on pulsatile GnRH stimulation, GnRH frequency regulation of differential LH and FSH secretions, and distinct pulse‐ and surge‐modes of GnRH release, among many others. The clinical ramifications were equally profound, prompting use of pulsatile GnRH therapies to treat hypothalamic hypo‐gonadotropism, and non‐pulsatile GnRH agonist treatments to suppress gonadotropin secretions in precocious puberty or prostate cancer therapies. And of course, there are many discoveries to come! These will include a deeper understanding of the molecular and cellular mechanisms by which the activity of arcuate Kiss1 neurons – likely KNDy neurons – are initiated and synchronized, and in turn how the network is regulated by both peripheral and central physiological signals. I am also certain that drug development will continue apace to capitalize on these discoveries to provide new treatments for reproductive disorders, such as polycystic ovary syndrome.

### 2.5 Tony Plant

The recognition during the first decade of this century of the primacy of kisspeptin in the upstream control of the GnRH neuron. In my view this is indisputable, and I would be surprised if others thought differently. Not only was this a major conceptual breakthrough but it also acted to rekindle interest (including that of Institutional support) in the study of GnRH and the HPG axis: a field of neuroendocrinology that for me had begun to languish somewhat. However, the time has now come to return to a more holistic approach to understanding the control of the GnRH neuron by establishing how other neural factors (including classical and nonclassical transmitters, neuropeptides and glial signals), many previously implicated in the regulation of GnRH secretion, are to be incorporated into our current kisspeptin paradigms.

### 2.6 Pam Mellon

Other than the profound initial discovery of this neuroendocrine decapeptide by Roger Guillemin and Andrew Schally, the main breakthroughs have been cloning of the GnRH gene and its receptor, establishing the very unusual developmental migration of the GnRH neuron from the olfactory placode to the hypothalamus, discovery of the phenomenon of the rapid pulsatile release and downregulation of the receptor, development of GnRH agonists and antagonists and their utilization in cancer treatment, and, more recently, regulation of GnRH neurons by kisspeptin and elucidation of many of the genes that are mutated in Kallmann Syndrome and idiopathic hypogonadotropic hypogonadism.

## 3 WHAT OBSTACLE WAS THE HARDEST TO OVERCOME IN REPRODUCTIVE NEUROENDOCRINOLOGY DURING THIS TIME?

### 3.1 Bill Crowley

Since the 1977 Nobel Prize was given for the discovery of GnRH and its potent agonist analogs, most scientists were naturally quite invested in the use of the GnRH analogs as agonists. Consequently, it was initially quite difficult to convince the scientific world that, when used in high doses, these GnRH agonists could paradoxically induce a highly selective and reversible homologous desensitization of the GnRH receptor that was quite safe and reversible and hence could be of great therapeutic value in removing the influences of gonadal sex steroids in those clinical circumstances where their ablation was associated with beneficial effects. The article describing this in men with isolated GnRH deficiency was initially rejected by the *New England Journal of Medicine* and only when it was demonstrated to be dramatic in its beneficial effects in children with isosexual precocious puberty in 1980 they eventually published it. As the vast majority of children with precocity are female (5–9:1 sex ratio); 40% of these girls were victims of childhood molestation; and GnRH agonist dramatically decreased this effect, eventually the “gospel” of GnRH agonism as the beneficial effect of long term “GnRH agonist” therapy was replaced by their functional antagonism. The eventual development of true GnRH antagonists did not occur for several decades and then their safety was not as well established as is GnRH agonists.

### 3.2 Bob Goodman

This is a difficult question as there were several, but I would pick as one of the major ones determining the physiological concentrations and patterns of GnRH, since this had to be done with samples of hypophysial portal blood from unanesthetized animals. [Correction added on 15 June 2022 after first online publication: In‐text citation of Wade 1981 was deleted from this paragraph.]

### 3.3 Jon Levine

The advent of genetic engineering in mice provided new avenues to study GnRH biology, and much progress was made using transgenics bearing reporter genes, gene knock‐outs, and gene knock‐ins – much progress up to a point, that is. While methods were developed to measure GnRH release and LH pulsatility in monkeys, sheep, and rats, the use of these approaches in mice was an obstacle that could not be overcome until recently, at least with respect to the LH pulsatility. We struggled to measure GnRH release profiles in mice by microdialysis, but it proved to be a bridge too far. Thus, for many years the reproductive neuroendocrine phenotypes of genetically engineered mice remained incomplete, limiting the power of observations made in these animals.

### 3.4 Pam Mellon

GnRH neurons are so limited in number, scattered around the hypothalamus, and elongated that they are relatively inaccessible for molecular and transcriptional studies in vivo. In the 1980s, new technologies were invented to express exogenous genes in mice and the GnRH gene was identified and cloned. Using the upstream regulatory region of the rat GnRH gene cloned onto the SV40 T antigen oncogene, we created transgenic mice that developed tumors of the GnRH neuron. Though the mice were all infertile and most died young, one of the tumors was cultured in vitro to create GnRH neuronal cell lines, in particular the GT1‐7 cell line. This cell line has allowed deep investigations into the molecular basis of regulation of the GnRH gene establishing many of the key transcription factors that directly regulate the GnRH gene. These have been subsequently validated in knockout and Cre/LoxP mouse models as crucial to GnRH neuron birth, migration, survival, and gene expression.

Similarly, the target cell of GnRH, the pituitary gonadotrope, is a small, scattered population of cells in the anterior pituitary, difficult to purify and thus hard to study at the molecular level. Using the same strategy, we were able to create cell lines representing several stages of gonadotrope development. In particular, the LβT2 mouse pituitary cell line produces luteinizing hormone (LH) and follicle‐stimulating hormone (FSH), as well as the GnRH receptor. The LβT2 cell line responds to pulsatile GnRH by preferentially secreting LH in response to fast pulses and FSH in response to slow pulses, as is seen in vivo. These cell lines allowed cloning of the GnRH receptor gene and characterization of many transcription factors and signaling pathways through which GnRH regulates the gonadotrope.

### 3.5 Ei Terasawa

One of the most important obstacles was the fact that GnRH neurons are scattered widely in the septum, preoptic area and basal hypothalamus and do not form discrete hypothalamic nuclei, unlike oxytocin and vasopressin neurons. As I stated above, the discovery of the GnRH molecule clarified the issue by eventually allowing for identification of GnRH neurons among numerous other neurons and glia. However, there was a huge competition for claiming the discovery. Young readers can find the story in the book entitled “The Nobel Duel” written by Nicholas Wade (Anchor Press/Doubleday, 1981). The competition was not only between Andrew Schally and Roger Guillemin, but also included Donald McCann, although this was not mentioned in the book. At that time, I heard that McCann was very depressed after learning he was not included as a recipient of the Nobel Award. [Correction added on 15 June 2022, after first online publication: Section 3.5 has been inserted.]

## 4 WHAT DO YOU THINK HAS BEEN THE MAIN BREAKTHROUGH OF THESE 50 YEARS OF GnRH RESEARCH? AND WHAT OBSTACLE WAS THE HARDEST TO OVERCOME IN REPRODUCTIVE NEUROENDOCRINOLOGY DURING THIS TIME?

### 4.1 Iain Clarke

Undoubtedly, this was the identification of kisspeptin as a regulator of GnRH secretion. For many years, I and others sought to “close the loop” of the hypothalamic‐pituitary‐gonadal axis, defining the way that sex steroids regulate GnRH cells. This was an important issue, given that GnRH cells do not express estrogen receptor‐α (or receptors for other gonadal steroids), even though the secretion of GnRH is regulated by gonadal steroids. Kisspeptin is now recognised as a major regulator of GnRH secretion. Nevertheless, other neuronal systems in the brain also express sex steroid receptors and either project directly to GnRH neurons or act via intermediate neurons to regulate GnRH neurons; the excitement over the role of kisspeptin has deflected attention to these. In particular, significant populations of noradrenergic and serotoninergic cells in the mid/hind brain are steroid responsive and probably play an integral role in the control of reproduction. As to how sex steroid feedback to GnRH neurons is integrated through a variety of neuronal circuits remains a major challenge.

## 5 IF YOU WERE ACTIVE AT THE TIME OF THE GnRH DISCOVERY, HOW HAS THE FIELD EVOLVED COMPARED TO YOUR EXPECTATIONS BACK THEN?

### 5.1 Iain Clarke

I commenced my research career in the 1970s, at a time when a variety of species were studied. In particular, a number of excellent laboratories throughout the world conducted research in domestic animals, which were large enough to allow serial measurement of hormones. Work in ungulates was seminal in deciphering the operation of the hypothalamic‐pituitary‐gonadal axis, especially in the female. This was because the relatively long estrous cycles of such species were more comparable to that of humans than the 4–5 day cycles of rodents. Furthermore, the endocrinology of the cycle differs between species, an example being the support of luteal function in rodents versus ungulates; in rats and mice, prolactin has an important role, whereas this is not the case in species such as sheep in which LH is the main factor driving luteal function. Importantly, the estrous cycle of rats and mice is tightly regulated by photoperiod, which is not the case in most other species. With the advent of transgenics, there was a shift towards work in mice, with a decline in work on other species. Sadly, this has led to a somewhat myopic view of how GnRH neurons are regulated, and the current literature rarely refers to work in nonrodent species. Accordingly, we are probably missing important facets of reproductive function. A classic example of how work in disparate species led to important findings is the story of mountain and prairie voles and the control of reproductive behavior. Although this is not directly linked to GnRH function, it serves to indicate that important discoveries may be made by working in a variety of species. Whilst knowledge has been vastly expanded by the use of rodent species and transgenics, work in other species could also contribute to our understanding of reproductive function in future.

### 5.2 Bill Crowley

I do not think any of us really anticipated the enormous impact that sequencing the human genome and identifying mutations in several human disease conditions would have on the science in this area. These human genetic studies have demonstrated that, given the marked species specificity of reproduction, it was now possible to conduct primary GnRH experimentation in human disease models. This revolution has had a major impact on research in this area. That said, scientific truths in the area of GnRH that are derived from humans always require validation and experimental validation and testing in vitro and in vivo in appropriate animal and cellular models. So the importance of basic scientists in this area has only grown except that now they know they are working on relevant problems when they have originated from human‐based research. As a leading investigator in this field, Allan Herbison said in an after‐dinner speech in Spain at a GnRH meeting: “When I think of all the time I wasted applying virtually every chemical in the Merck Manual to GnRH neurons, all too little or to no avail, I now realize that I should have simply sat down and had a beer and conversation with the clinical investigators in this area who were doing genetic studies.” Pretty good quote to summarize what I had been telling him for some time.

### 5.3 Bob Goodman

I would say that the evolution from systemic physiology, that relied on using RIAs and physiological replacement models that mimicked normal hormone levels to neuroscience that requires more sophisticated approaches.

### 5.4 John Marshall

In 1969, I began an endocrine fellowship at the Royal Post Graduate Medical School in London. My project was to develop LH/FSH assays which required preparation of all reagents (purified human LH was a gift from Ann Hartree in Cambridge); antibody was produced in rabbits and gamma globulin for a second antibody was obtained by exsanguinating tuberculous Guinea pigs! The assay was one of the first in Europe able to measure normal human plasma values and we initiated studies of human gonadotropin physiology. In late 1970 imagine the excitement of a young fellow when I received a call from a representative of Hoechst (who had provided funding to Andrew Schally's research): “Would I like a supply of synthetic GnRH to use in human studies?” This timing was most propitious and allowed us to initiate a multitude of studies in humans—role of GnRH in normal human physiology, puberty, ovulatory cycles, delineation of steroid feedback, GnRH self‐priming action—studies which contributed to much of our present understanding of human physiology.

### 5.5 Ei Terasawa

Before the discovery of GnRH molecule, specific structures in the brain controlling reproductive function were called tonic (MBH) and phasic (preoptic area) centers. This was based on studies such as electrical stimulation, lesions and knife cuts of portions of the preoptic area and basal hypothalamus, as well as perinatal sex steroid administration examining morphological changes related to sexual differentiation of the brain. At that time, the preoptic area‐hypothalamus was viewed as a big black box. The discovery of GnRH molecule led to clear insights as to where neurons expressing GnRH were distributed. I remember that in the rat brain GnRH perikarya were found in the preoptic area, but in the MBH only GnRH fibers were seen. Why? A clear answer to this question was not made until the discovery of kisspeptin molecule (and perhaps the discovery of NKB molecule), which are also indispensable for GnRH/LH release and subsequent studies of neurons expressing kisspeptin in the MBH and AVPV in the preoptic area. In addition, findings in the sheep MBH, demonstrating the colocalization of kisspeptin, NKB, and dynorphin in a group of cells, were made that would be important for pulsatility of GnRH release.

## 6 WOULD YOU HAVE CHANGED ANYTHING IN YOUR CAREER STUDYING GnRH KNOWING WHAT WE KNOW TODAY? FOR EXAMPLE, TIME SPENT ON FACTORS THAT TURNED OUT TO BE LESS RELEVANT FOR GnRH PHYSIOLOGY OR MISSED OPPORTUNITIES?

### 6.1 Bill Crowley

Not really. Because of our nearly exclusive focus on human genetics in the GnRH field so early on, virtually all the discoveries we made were guaranteed to be relevant to humans. Therefore, we did not waste any time on findings that are not relevant.

### 6.2 Jon Levine

Since my earliest days as a neuroendocrinologist, I have been interested in the mechanisms that mediate feedback actions of gonadal steroids on GnRH and LH release. At that time, the field was somewhat preoccupied with identifying the neurotransmitter cell groups that regulate GnRH release and characterizing the role that these neuronal groups might play in conveying the positive and/or negative feedback actions of estrogens and progesterone in females, and testosterone in males. The hunt for targets of estradiol became particularly intense with the publication by Donald Pfaff's group that found a paucity of estradiol‐concentrating GnRH neurons. Like many other laboratories, we completed many studies that implicated neuropeptide Y, opioid peptides, excitatory amino acids, etc., in steroid feedback mechanisms. I don't doubt that these neurotransmitters mediate some fraction of physiological signals to GnRH neurons, yet their importance in steroid feedback seems much diminished in light of the preeminent role that we now ascribe to kisspeptin neurons. Looking back on this work, I would have given more weight to the observations that only small fractions of some of these neurotransmitter cell groups express steroid receptors and focused more on other roles that these other neuronal groups may have played in physiological regulation of GnRH neurons.

### 6.3 Ei Terasawa

No, I would not have changed anything. I thought that, given the situation, there were always so many exciting projects. I enjoyed the process of new discoveries. [Correction added on 15 June 2022 after first online publication: Section 4.2 was shifted here as Section 6.2, and Section 6.2 was changed to Section 6.3.]

## 7 WHAT DO YOU CONSIDER THE MAJOR REMAINING UNANSWERED QUESTION(S) REGARDING GnRH NEURONS IN HEALTH AND/OR DISEASE? WHAT ARE MAJOR CHALLENGES OR OBSTACLES TO ANSWERING THAT QUESTION(S), AND WHAT WILL BE NEEDED FOR THAT TO HAPPEN?

### 7.1 Iain Clarke

We published a paper recently to show that glucagon like peptide‐1 is a potent stimulator of GnRH secretion, acting at the level of the median eminence. This raises a number of questions. First, how are gut secretions involved in the regulation of GnRH secretion in the wider sense? Second, the action of such a peptide at the level of the median eminence begs the question as to how it exerts its effect. It seems most probable that classical ligand binding is required but receptor expression within the median eminence is not apparent. The elegant descriptions of dendrons by the Herbison group are a major advance in our understanding of how GnRH secretion is controlled but much more needs to be done on mechanisms of control beyond the GnRH cell body. This should be a major area of focus in the future, bearing in mind that the projections into the neurosecretory zone of the median eminence differ between species. Work on larger animal species should proceed in parallel with work on rodent species.

### 7.2 Bill Crowley

To date, almost all genetic studies in the GnRH field have been made in families affected by powerful single Mendelian genetic loci. However, with the availability of ever‐larger human population studies (e.g., the UK Biobank's 500 k cohort), it is now possible to determine the roles of GnRH‐related disease‐causing loci in the complex genetic trait genetic arena. These studies will now reveal an ever‐increasing list of GnRH‐related genes that are contributing to the skewed sex ratios that are present in many (if not most) common medical diseases. This new area should be a vast arena in which to examine the broader role of GnRH‐related genes in the complex genetic architectures of many common diseases. Thus, the future is rich with opportunities as (1) whole genome sequencing costs continue to drop reaching $100/person very soon; (2) whole genome sequencing becomes the test of choice in all newborns to determine the presence of all genetic loci that can potentially contribute to diseases at birth (already started in the UK); and the rapid ability to mine ever larger populations and databases efficiently becomes the norm rather than the exception. This future is bright indeed.

### 7.3 Bob Goodman

I think one of the major remaining issues is distinguishing between neural inputs that are major regulators of GnRH secretion and those that can influence this system but are of minor physiological significance. This is a difficult problem because the relative importance of these inputs will vary depending on the external and internal environment. I personally think that a major challenge is the movement to the exclusive use of mice as a preclinical model. Given the major species differences in reproductive neuroendocrinology, there are clear questions about the relevance of this model to human reproductive function. Nevertheless, because of current sophisticated experimental techniques available with transgenic mice this trend appears to be accelerating and this obstacle is unlikely to be addressed.

### 7.4 John Marshall

I believe the major unanswered question is what are the precise mechanisms of the GnRH pulse generation system and how is it controlled by the CNS? Is the KNDy neuron system the pulse generator or an intermediate messenger from higher signals which control other clock and cyclical functions in the body? How is this pulse generator system influenced by interactions with other physiological systems—reduced bodyweight, calorie balance and all serious illness result in a marked slowing or loss of GnRH pulses and particularly in females, the reproductive system is a bellwether for general health of the whole animal. Elucidation of this regulatory systems will be complex, involving both local and distant interactions within the CNS and delineation of their nature will probably require developing the abilities to measure central interactions between different physiological regulatory systems. This is a major challenge, but solutions would allow interventions in many disorders and our ability to monitor the GnRH signal peripherally may provide insights into intersystem CNS interactions, probably applicable to other regulatory systems.

### 7.5 Tony Plant

The neurobiology underlying the dampening of pulsatile GnRH release from infancy until the onset of puberty that guarantees the relative quiescence of the prepubertal gonad in boys and girls. Three questions must be answered. (1) What is the nature of the switch (off switch) that leads to a decrease in GnRH drive to the pituitary‐gonadal axis during infancy. (2) What are the cellular and molecular components of the neurobiological brake that appears to be imposed on the kisspeptin GnRH pulse generator in the infundibular nucleus during childhood and juvenile development. (3) Is the onset of puberty after a protracted delay following infancy triggered by throwing the “off switch” of infancy into reverse? A major obstacle to answering these questions is the lack of an appropriate nonhuman paradigm that is genetically tractable. The last part of the question is the most difficult to address, and I do not have a definitive answer—maybe a quantum leap in development of methodologies required to noninvasively and continuously monitor temporal changes in activity of, and between, neuronal nuclei and other defined regions of the primate brain!

### 7.6 Ei Terasawa

First, as I wrote in my article for this special issue, the neural substrates responsible for “central inhibition” of GnRH neurons prior to puberty onset in primates is a critically important question, yet a full answer is not yet available. Second, we do not yet know the entire composition of the GnRH pulse generator and how it works. KNDy neurons are one of the key components, but recent work indicates that nitric oxide also appears to be involved, and we have observed release of NPY is pulsatile and NPY pulses are synchronous with GnRH pulses. Additionally, GABA and glutamate neurons are intimately involved in the regulation of GnRH, but where are they placed in between KNDy and GnRH neurons?

Third, the precise cellular and molecular mechanisms of GnRH neurodegeneration in the olfactory placode, as well as migration of surviving cells into the preoptic area and basal hypothalamus has been understudied. Because of this, there are few treatment tools for patients with idiopathic/congenital hypogonadotropic hypogonadism. Finally, during the adolescent period, major reorganization of the neural circuit of the brain occurs. This can be either steroid‐independent and/or steroid‐dependent, but proper timing of puberty onset, which is regulated by GnRH neurons, is one of the most important developmental events in human life. Presently, we have little knowledge between the start of GnRH release (onset of puberty) and the full maturation of the brain.

## CONFLICT OF INTEREST

The author has nothing to conflict of interest.

### PEER REVIEW

The peer review history for this article is available at https://publons.com/publon/10.1111/jne.13140.

## Data Availability

Data sharing is not applicable to this article as no new data were created or analyzed in this study.
